# The nexus between improved water supply and water-borne diseases in urban areas in Africa: a scoping review

**DOI:** 10.12688/aasopenres.13225.1

**Published:** 2021-05-28

**Authors:** Nyamai Mutono, Jim A Wright, Henry Mutembei, Josphat Muema, Mair L.H Thomas, Mumbua Mutunga, Samuel Mwangi Thumbi

**Affiliations:** 1 Wangari Maathai Institute for Peace and Environmental Studies, University of Nairobi, Nairobi, Kenya; 2 Washington State University Global Health Program - Kenya, Nairobi, Kenya; 3 Centre for Epidemiological Modelling and Analysis, University of Nairobi, Nairobi, Kenya; 4 Geography and Environmental Science, University of Southampton, Southampton, UK; 5 Department of Clinical Studies, Faculty of Veterinary Medicine,, University of Nairobi, Nairobi, Kenya; 6 Institute of Tropical and Infectious Diseases, University of Nairobi, Nairobi, Kenya; 7 Paul G Allen School for Global Animal Health, Washington State University, Pullman, USA; 8 Institute of Immunology and Infection Research, University of Edinburgh, Edinburgh, UK

**Keywords:** water sufficiency, waterborne diseases, urban Africa, review

## Abstract

**Background:** The sub-Saharan Africa has the fastest rate of urbanisation in the world. However, infrastructure growth in the region is slower than urbanisation rates, leading to inadequate provision and access to basic services such as piped safe drinking water. Lack of sufficient access to safe water has the potential to increase the burden of waterborne diseases among these urbanising populations. This scoping review assesses how the relationship between waterborne diseases and water sufficiency in Africa has been studied.

**Methods:** In April 2020, we searched the Web of Science, PubMed, Embase and Google Scholar databases for studies of African cities that examined the effect of insufficient piped water supply on selected waterborne disease and syndromes (cholera, typhoid, diarrhea, amoebiasis, dysentery, gastroneteritis, cryptosporidium, cyclosporiasis, giardiasis, rotavirus). Only studies conducted in cities that had more than half a million residents in 2014 were included.

**Results:** A total of 32 studies in 24 cities from 17 countries were included in the study. Most studies used case-control, cross-sectional individual or ecological level study designs. Proportion of the study population with access to piped water was the common water availability metrics measured while amounts consumed per capita or water interruptions were seldom used in assessing sufficient water supply. Diarrhea, cholera and typhoid were the major diseases or syndromes used to understand the association between health and water sufficiency in urban areas. There was weak correlation between the study designs used and the association with health outcomes and water sufficiency metrics. Very few studies looked at change in health outcomes and water sufficiency over time.

**Conclusion:** Surveillance of health outcomes and the trends in piped water quantity and mode of access should be prioritised in urban areas in Africa in order to implement interventions towards reducing the burden associated with waterborne diseases and syndromes.

## Introduction

The sub-Saharan Africa (SSA) has experienced the highest annual urban population growth rate (more than 3.5%) in the world ^1^. However, the growth of urban infrastructure has been slower, leading to populations without access to adequate resources including water services, health facilities, and housing ^2, 3^.

Globally, it is estimated that one in every two people will be living in water stressed areas by 2025 increasing the challenge of water supply ^4^. As of 2017, only half of the population residing in urban areas in SSA had access to improved water sources which included piped, boreholes, protected wells or springs, rainwater or packaged water ^5^. However, going by The World Bank categorisation of piped water as the only major source of improved water in urban areas in SSA ^6^, only 56% (230 million people) residing in urban areas in this region have access to clean water ^7^.

More than half a million deaths in SSA have been attributed to diarrheal diseases, with water contamination being one of the key risk factors ^8^. The global enteric multicenter study identified *Escherichia coli*, *Cryptosporidium*, *Aeromonas* spp, *Shigella* spp and *Entamoeba histolytica* to be associated with increased risk of death among children younger than 24 months with moderate-to-severe diarrhea ^9^. Due to their high burden, several waterborne diseases including cholera, bloody diarrhea and typhoid are included in the Integrated Disease Surveillance Strategy used in most African countries to improve countries speed of detection and response to public health threats ^10^.

The United Nations Sustainable Development Goals (SDGs) 3, 6 and 11 that focus on good health and wellbeing of populations; clean water and sanitation; and sustainable cities and communities directly or indirectly address this problem associated with rapid urbanisation in SSA ^11^. The African Union Agenda 2063 aspires to have an African continent that is based on inclusive growth and sustainable development ^12^. To reduce the burden of waterborne diseases in the context of an urbanising population, a good understanding of the relationship between water and these health outcomes is required.

Previous reviews have focused on water quality ^13, 14^, water availability ^15, 16^ and the reallocation of water from rural to urban regions in Africa ^17^. Other reviews have also focused on the environmental determinants of waterborne disease outbreaks in Africa ^18^, the link between waterborne diseases and water resource development in Africa ^19^ and climate change globally ^20^. To ensure a medium level of health concern, an access of at least 50 litres per person per day is required ^21^. However, there is a gap on insufficient access to piped water (less than 50 litres per person per day) in urban areas in Africa and the association with waterborne diseases and syndromes in the African continent.

Here, we conduct a scoping review to assess the link between sufficient access to piped water supply and waterborne diseases and syndromes in African cities. Specifically, we answer the following questions: i) How has the relationship between waterborne diseases and piped water sufficiency been studied in Africa? ii) Are there under-utilised study designs, under-studied metrics of water sufficiency or under-studied syndromes or waterborne diseases?

## Methods

### Literature search methods

This scoping review was conducted following the Joanna Briggs Institute methodology guidance for scoping reviews ^22^ and the preferred Reporting Items for Systematic Reviews and Meta-Analyses (PRISMA) extension guidelines for conducting scoping reviews ^23, 24^. Briefly, this approach involves: i) conducting a systematic literature search to identify articles that meet the inclusion criteria, ii) assessing the relevance of the articles to the study question(s), iii) assessment of the full text articles iv) data extraction and synthesis. The scoping review protocol for this study is published and available ^25^.

### Information sources and search strategy

In April 2020, literature searches were undertaken in the following four electronic databases: Embase, MEDLINE, Web of Science and Google Scholar (first 500 papers). These have been identified as the optimal combination of databases that would guarantee adequate and efficient coverage of studies for literature searches ^26^. The exact dates when searches were conducted can be found in Table A1.

**Table A1.** Exact dates when the searches were run in the databases.

Database	Date
Embase	13 ^th^ April, 2020
MEDLINE	9 ^th^ April, 2020
Web of Science	9 ^th^ April, 2020
Google Scholar (first 500)	10 ^th^ April,2020

The search strategy consisted of a two-step process. The first step involved carrying out a limited search in MEDLINE, Embase and Web of Science databases to analyse the text words and index terms that are used to describe the articles. The second step included a keyword search in all four databases; index terms were also used. The search terms that were used in the study can be seen in Table 1. The search terms include a combination of names of all African cities that have a population of at least half a million residents as of 2014, as outlined in the protocol ^25^, and terms representing the exposure (insufficient piped water supply) and outcome (waterborne diseases and syndromes). The study focused on publications that were written in the English or French language.

**Table 1.** Search terms that were used to select studies from the different electronic databases.

Parameter	Search terms
Population	Huambo OR Luanda OR Cotonou OR “Abomey-Calavi” OR “Abomey Calavi” OR Ouagadougou OR Bobo-Dioulasso OR “Bobo Dioulasso” OR Bunjumbura OR Younde OR Yaounde OR Douala OR Bangui OR Ndjamena OR Brazaville OR Pointe-Noire OR “PointeNoire” OR Abidjan OR Bouake OR Kinsasha OR Cairo OR “Al Qahirah” OR Al-Qahirah OR Alexandria OR “Al-Iskandariyah” OR “Al Iskandariyah” OR “Port Said” OR “Bur Said” OR “Addis Ababa” OR Libreville OR Banjul OR Accra OR Kumasi OR Conakry OR Nairobi OR Mombasa OR Monrovia OR Antananarivo OR Lilongwe OR “Blantyre-Limbe” OR “Blantyre Limbe” OR Bamako OR Nouakchott OR Casablanca OR “Dar-el-Beida” OR “Dar el Beida” OR Rabat OR Nampula OR Tetouan OR Fes OR Marrakech OR Tangier OR Tanger OR Maknes OR Meknes OR Agadir OR Maputo OR Matola OR Niamey OR Lagos OR Kaduna OR Akure OR Kano OR Abuja OR Aba OR Kigali OR Dakar OR Freetown OR “Cape Town” OR Durban OR Pretoria OR “Port Elizabeth” OR Bloemfontein OR “Dar es Salaam” OR Arusha OR Mbeya OR Lome OR Kampala OR Kitwe OR Lusaka OR Harare OR Bulawayo OR “Benin City” OR Enugu OR Ibadan OR Ikorodu OR Ilorin OR Jos OR Maiduguri OR Nnewi OR Onitsha OR Oshogbo OR Owerri OR “Port Harcourt” OR Sokoto OR Umuahia OR Oyo OR Warri OR Zaria OR Hargeysa OR Merca OR Mogadishu OR Muqdisho OR Johannesburg OR Soshanguve OR Vereeniging OR Khartoum OR “Al-Khartum” OR “Al Khartum” OR Nyala OR Safaqis OR Tunis OR Mwanza OR Zanzibar OR Ndola OR Algiers OR “El Djazair” OR Wahran OR Oran OR Bukavu OR Kananga OR Kisangani OR Lubumbashi OR “Mbuji-Mayi” OR “Mbuji Mayi” OR Tshikapa OR Djibouti OR “Al-Mansurah” OR “Al Mansurah” OR “As- Suways” OR “As Suways” OR Asmara OR “Sekondi Takoradi” OR Banghazi OR Misratah OR Tarabulus OR Tripoli
	*AND*
Exposure	water AND (scarc* OR intermittent OR break* OR ratio* OR deficit OR deficien* OR unavailab* OR availab* OR continu* OR interrupt* OR stress OR supply OR sufficien* OR insufficien*)
	*AND*
Outcome	“water borne” OR “water-borne” OR cholera OR typhoid OR diarrhea* OR diarrhoea OR amoebiasis OR dysentery OR gastroenteritis OR cryptosporidi* OR cyclosporiasis OR giardiasis OR rotavirus

### Data screening

Once searches were complete, the title and abstracts were extracted from the articles. Duplicates were removed and three reviewers (NM, JM, MM) independently screened the study titles and abstracts using the following criteria:

1)   Studies that described the water sufficiency or water situation in cities with populations more than 500,000 in 2014

2)   Studies that focused on cholera, typhoid, amoebiasis, cyclosporiasis or giardiasis as diseases, dysentery, diarrhea or gastroenteritis as symptoms or cryptosporidium or rotavirus as etiological agents for diarrheal diseases ^27, 28^;

3)   Studies published in international scientific indexing (ISI) listed journals

Any inconsistencies between the three reviewers were discussed and a consensus was reached on whether to include or remove articles from the study.

### Study selection

Where available, the full text articles were obtained for all studies that met the inclusion criteria. Two reviewers (NM and MM) assessed and characterised the studies by analysing if they primarily targeted urban residents and had evaluated the relationship between a health outcome and a water sufficiency metric. The data extracted from this screening process were stored in an Excel spreadsheet.

### Data extraction, synthesis and presentation

Variables on author(s), study period, source of funding, geographical scope, study design, population inclusion criteria, sample size and statistical methodology used, and whether or not the study investigated a disease outbreak were extracted from the studies.

To understand piped water access and quality reported by the studies, we extracted information on the nature of the piped water supply, mode of accessing this piped water, measurement of the unit cost of water, the per capita daily water consumption, proportion of the population without access to piped water and water quality indicators from water samples collected for testing. The reported coping mechanisms employed to supplement water needs were also extracted. Information on the health outcomes studied and how diagnoses was made (self-reported, clinically diagnosed or culture confirmed) was also extracted from the articles. Table 2 provides a list of the variables extracted from the articles during the screening process.

**Table 2.** Description of variables that were extracted from the articles during full-text screening.

Variable	Description/ Example
**Study design**	
Study period	Year(s)
Geographical scope of the study	City/ cities where the study was conducted
Source of funding	Government sponsored/ philanthropic foundation/ research institute/ not sponsored
Study design	Cross-sectional individual/ cross-sectional ecological/ case control/ case series/ cohort
Population inclusion criteria	Households/ women/children/confirmed cases etc.
Sample size (people)	Number of respondents / households
Sample size	Number of water/ stool/ soil samples for testing
Outbreak investigation	Yes/No
**Statistical methodology used**	
Bivariate methods	Chi-square tests, Fischer tests etc.
Multivariate methods and	Linear models, logistic models etc. and confounders/ alternative transmission pathways / effect modifiers assessed
**Indicators of piped water sufficiency**	
Nature of piped water supply	Continuous/ scheduled interruptions/unpredictable interruptions
Mode of water access	Inhouse piped connection, shared tap at yard, public tap/water kiosk
Unit cost of water reported	Yes/No
Measurement of per capita daily water consumption	Yes/No
Proportion of population without access to piped water	Metric
Water quality indicators from water samples collected for testing	
- Faecal indicator organism test	e.g., total coliforms, Escherichia coli
- Dosage test for chlorine	e.g., Free chlorine residual test
- Pathogen tested for	e.g., *Klebsiella pneumoniae*, *Salmonella* spp., *Shigella* spp., *Pseudomonas aeruginosa*
- Consumer reported organoleptic water characteristics	e.g., smell, taste, visual appearance
- Laboratory or organoleptic field tests	e.g., electroconductivity, pH and turbidity
**Coping mechanism employed to** **supplement water needs**	
- Use of storage tanks	Yes/No
- Storage of water in households in containers, bottles etc.	Yes/No
- Installation of pumps for piped water where water pressure is low	Yes/No
- Collecting water from rivers/streams, shallow wells, rainwater	Yes/No
- Drilling of wells/boreholes	Yes/No
- Installation of hand pumps/electric pumps for groundwater	Yes/No
- Water treatment	Yes/No
- Purchasing water from vendors	Yes/No
- Purchasing water from neighbors	Yes/No
- Water recycling	Yes/No
- Illegal water connections	Yes/No
**Indicators of health**	
- Cholera	Self-reported/Clinically diagnosed/ laboratory confirmed
- Typhoid	Self-reported/Clinically diagnosed/ laboratory confirmed
- Amoebiasis	Self-reported/Clinically diagnosed/ laboratory confirmed
- Cyclosporiasis	Self-reported/Clinically diagnosed/ laboratory confirmed
- Giardiasis	Self-reported/Clinically diagnosed/ laboratory confirmed
- Dysentery	Self-reported/Clinically diagnosed/ laboratory confirmed
- Diarrhea	Self-reported/Clinically diagnosed/ laboratory confirmed
- Gastroenteritis	Self-reported/Clinically diagnosed/ laboratory confirmed
- Cryptosporidium	Self-reported/Clinically diagnosed/ laboratory confirmed
- Rotavirus	Self-reported/Clinically diagnosed/ laboratory confirmed

### Assessment of the study quality

We used the Strengthening the Reporting of Observational Studies in Epidemiology (STROBE) checklist to analyse the quality of the studies included in the scoping review ^29^. We assessed the studies based on whether the study objective was comprehensively stated, the study design, description of study location and dates of data collection were provided, provision of participant eligibility criteria and rationale given for sample size, explanation of how missing data was handled and how they controlled for confounders. No study was excluded based on it being poor quality.

### Connectedness of the study designs of the associations between water sufficiency and health outcomes

To understand the connectedness of the different study designs with the health outcomes and water sufficiency metrics and water quality, we used the principal component techniques ^30^. The main categories of the study designs employed in the selected publications were evaluated together with the health outcomes (self reported, clinically or culture confirmed) and a binary coding of assessment of water quality. The water sufficiency metrics were coded into either water access (mode of access, proportion with access, time/distance to water points) or water quantity (scheduled/ unscheduled interruptions, litres per person per day) categories. We carried out multiple factor analysis by grouping the study designs, health outcomes, water sufficiency metrics and whether water quality was assessed. We looked at the contributions of the first two axes and assessed the combinations of the variables that were connected, understudied and the outliers. The analysis was carried out using the FactomineR package in the statistical software R ^31, 32^.

## Results

### Study selection

**Figure 1.**
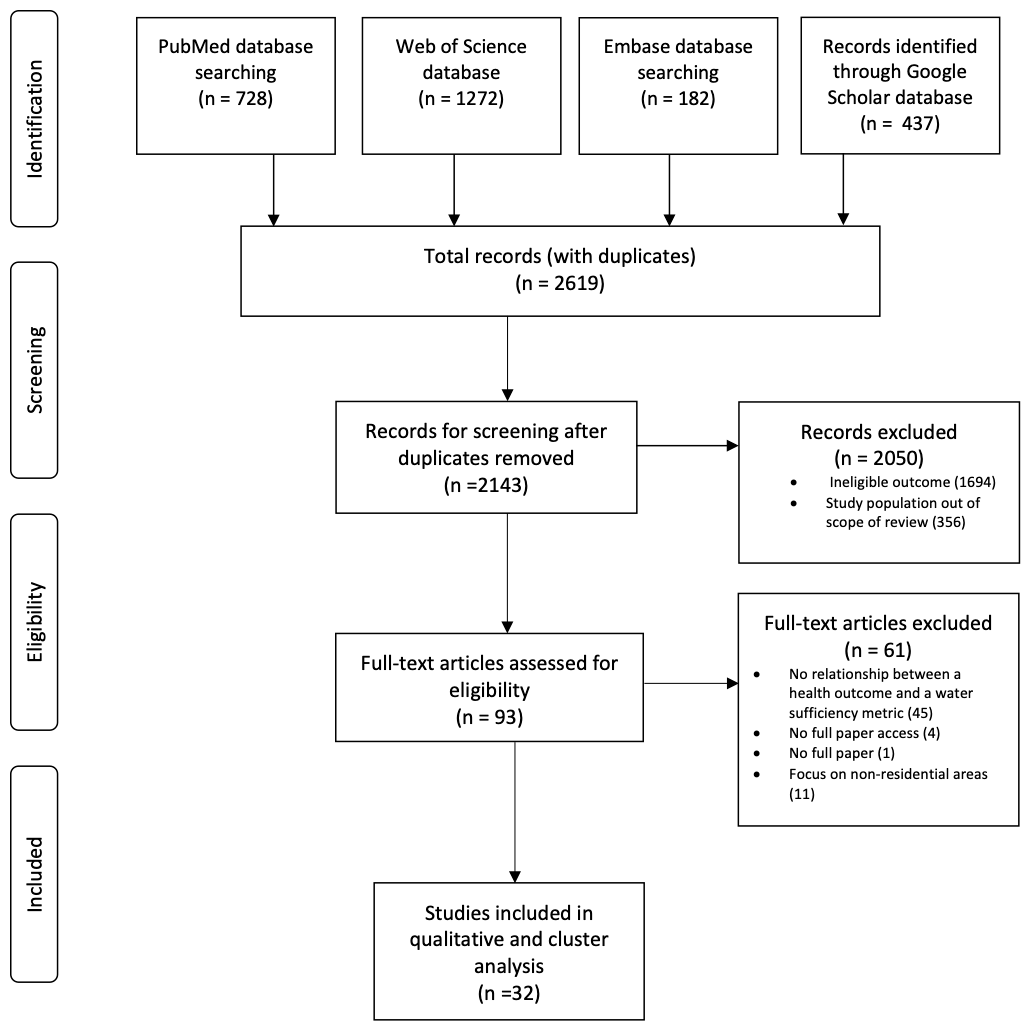
Flow diagram summarising the number of articles included at each review stage.

The initial database search revealed 3,099 articles. After removing duplicates, and assessing the abstracts for eligibility, 93 articles remained for full text review, with 32 of those studies meeting the inclusion criteria ( Figure 1).

### Quality of the studies

From our checklist, there were some strengths and weaknesses of the studies. All the studies had a clearly stated objective, study design and study location with date of data collection. The eligibility criteria of the study participants were also clearly stated by majority of the studies (n=31, 97%).

Three quarters of the studies reported on the statistical methods employed (n=24, 75%). Less than a third of the studies explained how the study size was calculated (n=8, 25%), the criteria used in choosing the quantitative variables (n=2, 6%) and how the studies controlled for confounders (n=9, 28%). None of the studies explained how they addressed missing data ( Table 3).

**Table 3.** Assessment of the quality of included studies.

Item	Parameter	Description	Criteria met N (% of studies)
1	Objective	Objective of the study comprehensively stated.	32 (100%)
2	Study design	The study design is clearly stated.	32 (100%)
3	Study setting	Study location and dates of data collection described.	32 (100%)
4	Participants	Eligibility criteria and selection method of participants declared.	31 (97%)
5	Sample size	Rational given for sample size.	8 (25%)
6	Statistical methods	All statistical methods are explicitly described.	24 (75%)
7	Variable justification	Explanation of the criteria used to choose the quantitative variables is included.	2 (6%)
8	Missing data	Explanation of how missing data was addressed is included.	0 (0%)
9	Controlling for confounders	Unadjusted estimates and confounder-adjusted estimates are provided.	9 (28%)

### Characteristics of the publications

**Figure 2.**
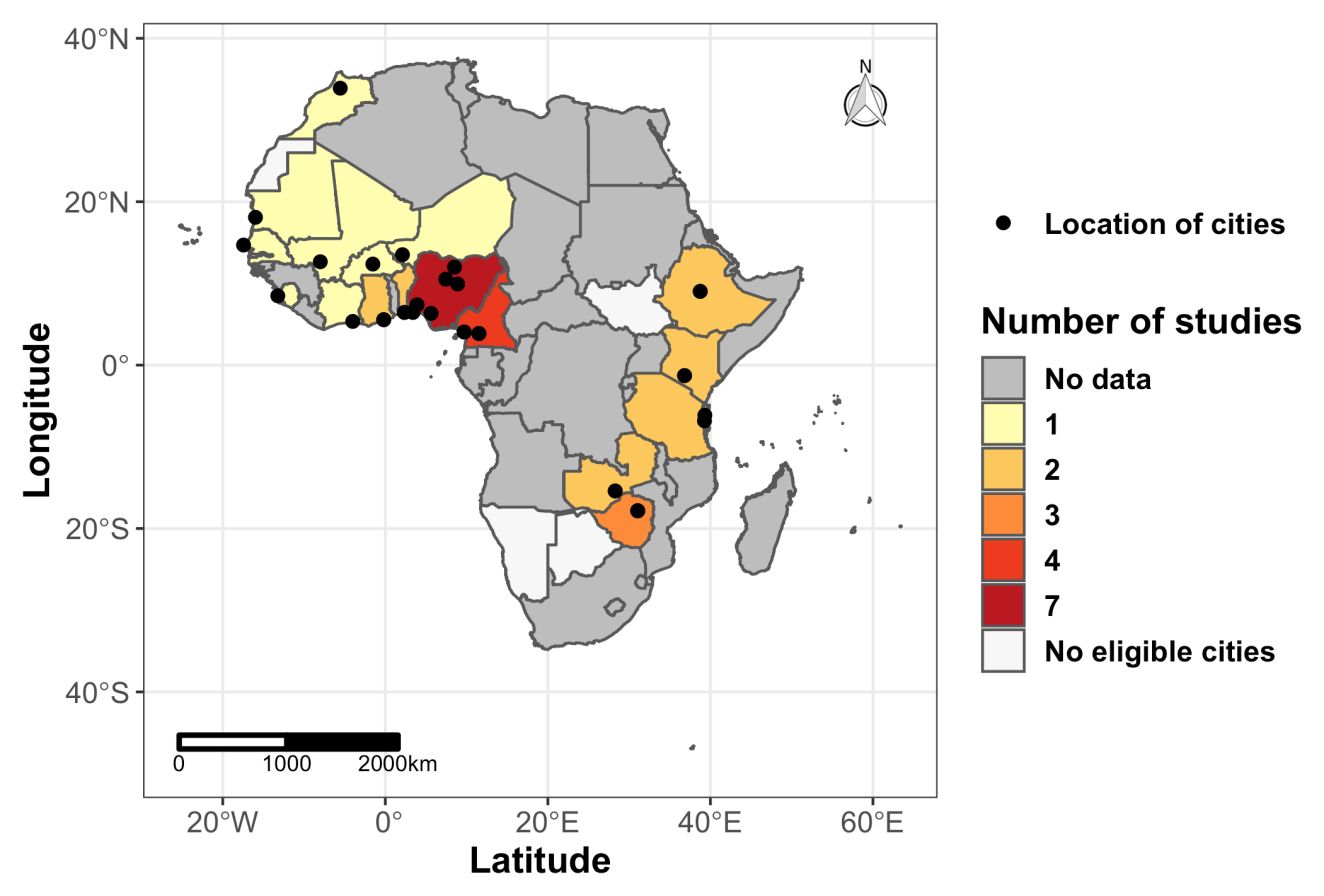
Geographical distribution of the studies and cities included in the scoping review Basemap source (shapefile): Database of Global Administrative Areas

A total of 32 articles that assessed the association of water sufficiency in urban areas and waterborne diseases and syndromes in SSA were published between 1998 and 2019. These studies focused on 24 cities in 17 countries across Western, Eastern and Southern Africa, with 22% (n=7) of the studies based in urban Nigeria ( Figure 2). Seven of the articles (22%) were conducted in informal settlements ^33– 40^. Nearly half the studies did not report the source of funding, with government and philanthropies supporting most of the studies that provided that information ( Table 4).

Half of the studies (n=16, 50%) employed cross-sectional individual level study design, and only six percent (n=2) used cohort study designs, with the rest utilising case-control or cross-sectional ecological designs ( Table 4). All these publications employed quantitative methods of data collection whereas only two publications (n=2, 6%) collected qualitative data to complement the quantitative data ^38, 41^. The studies’ target population included general households or respondents (n=17, 53%), confirmed cases or patients in hospitals being treated for waterborne diseases/syndromes (n=6, 19%), children below 10 years (n=6, 19%), women or mothers of infants (n=3, 9%) and HIV infected persons (n=3, 9%). The study subjects ranged from less than 100 (n=2, 6%) to more than 500 (n=11, 34%) and nearly a third of the articles (32%) were targeting outbreaks from cholera (n=9) or typhoid (n=1), which are epidemic-prone waterborne diseases ( Table 4).

**Table 4.** Characteristics of the 32 studies included in the scoping review.

Characteristic	No. of studies (% of included studies)	References
**Study period ^†^**		
≤2005	12 (38%)	37, 40, 42– 49
2006 – 2012	14 (43%)	34, 35, 38, 39, 42, 44, 50– 59
≥2013	8 (25%)	33, 36, 41, 60– 64
**Source of funding**		
Not reported	15 (47%)	34, 39, 40, 44– 48, 53, 55, 60, 61, 63– 65
Government departments/ agencies	6 (19%)	35, 36, 38, 43, 58, 59
Philanthropic foundations	5 (16%)	50, 52, 56, 57, 62
Research Institutes	4 (13%)	33, 42, 49, 51
Not sponsored	2 (6%)	41, 54
**Study design**		
Cross- sectional individual-level	16 (50%)	34– 36, 38, 40– 42, 49– 51, 53, 57, 60, 62
Case-control	9 (28%)	33, 39, 46, 55, 56, 58, 59, 64
Cross-sectional ecological	7 (22%)	37, 44, 47, 48, 52, 54
Cohort	2 (6%)	43, 61
Cross-sectional ecological and individual level	1 (3%)	45
Cross-sectional individual-level and case control	1 (3%)	63
**Population inclusion criteria ^†^**		
Households/ respondents	17 (53%)	34, 38– 41, 43– 45, 49, 52, 53, 55, 57, 59, 62– 64
Confirmed cases/ people visiting health facilities for treatment of waterborne diseases	6 (19%)	37, 45, 47, 48, 54, 61
Children/ infants	6 (19%)	33, 35, 42, 49, 51, 56
Women or mothers of infants	3 (9%)	35, 36, 40
HIV positive persons	3 (9%)	43, 36, 60
**Study population sample size**		
100	2 (6%)	34, 41
101–200	7 (22%)	37, 38, 40, 46, 54, 59, 62
201–300	7 (22%)	39, 43, 44, 53, 55, 60, 64
301–400	4 (13%)	45, 49, 57, 61
400–500	1 (3%)	58
>500	11 (34%)	33, 35, 36, 42, 47, 48, 50– 52, 56, 63
Study investigating an outbreak	10 (31%)	34, 37, 39, 47, 55, 57, 59, 61, 63, 64
**Statistical methodologies used (n=25) ^†^**		
Bivariate methods (chi-square tests, Fischer tests etc.)	17 (68%)	34– 36, 42, 43, 45, 50, 52– 54, 56– 59, 60– 62, 64
Multivariate methods (Linear models, logistic models etc.)	12 (48%)	33, 35, 36, 39, 40, 45, 51, 55, 56, 59, 62, 63
**Nature of piped water supply ^†^**		
Proportion with access to piped water	23 (72%)	34– 40, 42– 46, 48, 50– 53, 56, 58– 62
Water interruptions (scheduled/unpredictable)	8 (25%)	33– 35, 38, 39, 46, 53, 59
Per capita daily water availability	5 (16%)	33, 49, 51– 53
Cost / affordability of water metric	4 (13%)	35, 41, 52, 53
Time used/distance to water point	3 (9%)	51, 53, 56
**Samples collected ^†^**		
Water	19 (59%)	33, 34, 38, 41– 49, 54– 56
Stool	5 (16%)	55, 58, 59, 63, 64
Soil	1 (3%)	62
Hand rinse	1 (3%)	62
**Water quality indicators (n=19) ^†^**		
Faecal indicator organism test	17 (89%)	33, 34, 38, 41, 42, 44– 49, 52, 54– 56, 62, 63
Free chlorine residual test	7 (37%)	34, 44, 46, 48, 55, 56, 63
Laboratory/field tests organoleptic water characteristics	5 (26%)	41, 43, 44, 48, 59
Pathogen tests	5 (26%)	38, 42, 45, 49, 54
**Coping mechanisms employed ^†^**		
Collecting rainwater/ from rivers, streams, shallow wells etc.	22 (69%)	36– 42, 44, 45, 47– 49, 50– 56, 58, 60, 61, 64
Purchasing water from vendors	16 (50%)	34– 38, 43, 45, 46, 50– 53, 56, 59, 60, 64
Storing water in the households	11 (34%)	33– 35, 38, 43, 46, 51, 55, 56, 59, 62
Water treatment	8 (25%)	34, 40, 43, 45, 46, 50, 53, 55
Drilling wells/boreholes	3 (9%)	41, 48, 54
Purchasing water from neighbors	1 (3%)	41
Installing storage tanks in households	1 (3%)	41
Purchasing pumps for ground water	1 (3%)	41
Illegal water connections	1 (3%)	59
**Health outcomes- Self reported ^†^**		
Diarrhea	15 (47%)	33, 35, 36, 39– 43, 46, 50, 51, 55, 58, 60, 62
Cholera	4 (13%)	34, 53, 57, 63
Dysentery	3 (9%)	41, 50, 53
Typhoid	3 (9%)	41, 50, 53
**Clinically diagnosed ^†^**		
Cholera	8 (25%)	37, 39, 45, 47, 48, 54, 59, 61
Typhoid	4 (13%)	44, 45, 54, 61
Cryptosporidium	1 (3%)	38
Amoebiasis	1 (4%)	54
Diarrhea (uncategorised)	3 (9%)	44, 52, 54
Moderate to severe diarrhea	1 (3%)	56
Gastroenteritis	3 (9%)	44, 45, 54
Dysentery	3 (9%)	44, 45, 54
Rotavirus	1 (3%)	38
**Culture confirmed**		
Typhoid	1 (3%)	55
Cholera	1 (3%)	64
Cryptosporidium	1 (3%)	58

†A study appeared in more than one category

To understand the association between water and waterborne diseases and syndromes, the studies mainly used bivariate and multivariate methods of analysis. The common bivariate analysis methods used included the chi-square tests, Fisher tests, Wald tests and the correlation coefficient methods while the multivariate analysis methods included regression models (linear, logistic, random effects) and ANOVA models. The multivariate analysis models controlled for confounders/ effect modifiers in the analysis using independent variables which included source of water, type of water storage container, presence of water treatment, household hygiene and sanitation conditions, household characteristics which included size, income, employment, and presence of children ( Table 4). A study done by Machdar *et al* employed cost-effective analysis methods to assess the cost-effectiveness of interventions for reducing the disease burden from consumption of poor drinking water ^38^.

Piped water was mainly supplied by the utility companies to residents through inhouse connections, shared taps at compound or public taps/ water kiosks ^33, 36, 42, 50, 51, 60^. However, the publications reported piped water insufficiency through proportion of the study population that had access to piped water (n=23, 72%), scheduled/ unpredictable water interruptions (n=8, 25%), per capita daily water availability (n=5, 16%) and time used/ distance to the water point (n=3, 9%). Four articles reported piped water inequality through the mode of access (n=3, 9%) ^42, 50, 52^, quantity (n=2, 6%) ^38, 52^, cost (n=1, 3%) ^52^ and the scheduled water interruptions (n=1, 3%) ^38^.

The objective assessment of water safety was assessed by the studies via testing water samples (n=19, 59%). The water samples were collected from the dominant water points of the study population (n=9, 47%), water stored in the households (n=7, 37%), both dominant water points and stored water in the households (n=3, 16%) or hand rinse samples (n=1, 3%). Several studies assessed water contamination by testing for coliforms (n=17, 89%), effectiveness of measures of protecting water from contamination through testing for free residual chlorine (n=7, 37%), organoleptic characteristics of water by assessing turbidity and pH (32%, n=6) and presence of pathogens which included *klebsiella pneumoniae*, *staphylococcus aureus*, *pseudomonas aeruginosa*, among others (26%, n=5).

To complement their water needs, the study population employed coping mechanisms which included collecting rainwater/ water from rivers, streams or shallow wells (n=22, 69%), purchasing water either from vendors (n=16, 50%) or neighbors (n=1, 3%), storing water in the households (n=11, 34%), water treatment (n=8, 25%), drilling wells/ boreholes (n=3, 9%), installing storage tanks in households (n=1, 3%) and having illegal water connections (n=1, 3%) ( Table 4). Four of the studies reported a relatively higher cost in the purchased water as compared to the cost of water supplied by the utility companies ^35, 41, 52, 53^.

The publications focused on cholera (n=12, 38%), typhoid (n=8, 25%) and amoebiasis (n=2, 6%) as waterborne diseases, diarrhea (n=20, 32%), dysentery (n=7, 22%) and gastroenteritis (n=3, 9%) as symptoms and cryptosporidium (n=2, 6%) and rotavirus (n=1, 3%) as etiological agents of diarrheal diseases. The health outcomes were either self-reported, clinically confirmed or objectively assessed through collecting and culturing stool samples.

The most common self-reported waterborne diseases/syndromes included diarrhea (n=15, 47%), cholera (n=4, 13%), dysentery (n=3, 9%) and typhoid (n=3, 9%). The clinically confirmed health outcomes were cholera (n=8,25%), typhoid (n=4, 13%), cryptosporidium (n=1, 3%), amoebiasis (n=1, 3%), diarrhea (n=3,9%),moderate to severe diarrhea (n=1, 3%), gastroenteritis (n=3, 9%), dysentery (n=3, 9%) and rotavirus (n=1, 3%) while the culture confirmed health outcomes were typhoid (n=1, 3%), cholera (n=1, 3%) and cryptosporidium (n=1, 3%) ( Table 4). One study reported mortality as well as morbidity of waterborne diseases and syndromes ^61^. A comprehensive table containing the study characteristics can be found in Table B1.

**Table B1.** List of studies included in the review.

No	author(s)	Study period	Study design	Target population	Study population sample size	Rationale given for sample size	Sample type and size	Water insufficiency metric	Coping mechanism employed	Disease/ Syndrome studied (method of measuring) ^1^	Type of water tested	Water quality tests	Outbreak investigation	Analysis methods	Confounders/ effect modifiers/ other transmission pathways included in analysis
1	Degbey *et al.*(2011)	2008-2009	Cross- sectional ecological	Patients visiting health facilities	110	No	Water-110	NR	Digging wells, collecting water from alternative sources	Cholera (C), typhoid (C), amoebiasis (C), dysentery(C), diarrhea (C), gastroenteritis (C)	Water points	Faecal indicator organism test, pathogens test	No	Bivariate (Pearson chi-square tests; Fischer exact tests)	
2	Navab- Daneshmand *et al.* (2018)	2016	Cross- sectional individual	Households	142	Yes	Soil from outdoor location closest to the house entrance- 142; Water- 244 Hand rinse samples- 142	Proportion with running tap water	Water storage in household	Diarrhea (SR)	Stored water in household	Organoleptic water quality; Faecal indicator organism test	No	Multivariate (multiple regression models), bivariate (Pearson correlation coefficients)	Faecal indicator organism test result for soil, handwashing water, hands before washing, diarrhea incidence, number of assets owned, sanitation (presence of animals, toilet structure, toilet cleanliness, toilet location), household hygiene (presence of trash, presence of flies), Location of handwashing facility, presence of soap and water in handwashing facility, Organoleptic quality of stored water, type of opening of water storage container
3	Traore *et al.* (2013)	2008-2009	Cross- sectional ecological	municipalities	13,705	No	Water- 150	Proportion with access to piped water, L/P/D	Purchasing water, Collecting water from alternative sources	Diarrhea (C)	Water points	Faecal indicator organism test	No	Bivariate (Correlation coefficient)	Quality of water, incidence of waterborne disease/ syndrome
4	Sinyange *et al.* (2018)	2017-2018	Cross- sectional individual - KAP, Case- control	Households	267,205	No	Water-220, stool-4	NR	NR	Cholera (SR)	Water points	Faecal indicator organism test, free chlorine residual	Yes	Multivariate (NR)	Contact with a person with cholera, consumption of untreated water, gender
5	Ako *et al.* (2009)	1995-2006	Cross- sectional ecological	Households	300	No	Water-10	Proportion with access to piped water	Collecting water from alternative sources	Typhoid (C), diarrhea (C), amoebic dysentery (C), gastroenteritis (C)	Water points	Faecal indicator organism test, organoleptic water quality, free chlorine residual	No	NR	
6	Winter *et al.* (2019)	2016	Cross- sectional individual	women	550	No	N/A	Proportion with access to piped water	Purchasing water, Collecting water from alternative sources	Diarrhea (SR)	N/A	N/A	No	Bivariate (Pearson chi-square tests), multivariate (logistic regression)	Age, level of education, employed, has children, level of household income, type of toilet used during the day and at night, source of water, toilet hygiene and accessibility, WASH knowledge and practices
7	Sakijege (2019)	2018	Cross- sectional individual	households	32	No	Water-3	NR	Drilling boreholes, collecting water from alternative sources, purchasing water, storage tanks	Typhoid (SR), diarrhea (SR), dysentery (SR)	Water points	organoleptic water quality, Faecal indicator organism test	No	NR	
8	Muti *et al.* (2014)	2011	Case- control individual	respondents	230	No	Water-25 Stool-NR	NR	Water storage in households, water treatment, collecting water from alternative sources	Typhoid (CC), diarrhea (SR)	Water points	Free chlorine residual, faecal indicator organism test	Yes	Multivariate (NR)	Burst sewer pipe within 500 metres from home, typhoid contact at home, water from an alternative source, type of storage water container, boil drinking water
9	Oguntoke *et al.* (2009)	1999-2004	Cross- sectional ecological, cross- sectional individual	Patients visiting health facilities, households	350	No	Water-NR	Availability of piped water	Collecting water from alternative sources, purchasing water, water treatment, rainwater harvesting	Cholera (C), typhoid (C), dysentery (C), gastroenteritis (C)	Stored water in household	faecal indicator organism test, pathogen test	No	Bivariate (correlation coefficient) Multivariate (simple linear regression model)	Water treatment, level of income, household size
10	Dos Santos *et al.*, (2015)	2012	Cross- sectional individual	children under 10 years	702	No	N/A	L/P/D, Proportion with access to piped water, time spent to collect water	Purchasing water, water storage in household, rainwater harvesting	Diarrhea (SR)	N/A	N/A	No	Multivariate (logistic regression model)	Main source of drinking water, time spent in water collection, per capita water available, type of water storage container, use of rainwater, handwashing before eating, sex of household head, level of education of household head, livelihood of household head, economic status of household head, number of children in the household, type of sanitation
11	Essayagh *et al.* (2019)	2013-2016	Cohort	Typhoid confirmed cases	322	Yes	N/A	Proportion with access to piped water	Collecting water from alternative sources	Typhoid (C)	N/A	N/A	Yes	Bivariate (Wald Test)	
12	Barzilay *et al.* (2011)	2005	Cohort	HIV positive women	242 baseline and 187 followup visits	Yes	Water (baseline-242, followup visits- 187)	Proportion with an improved water supply	water storage at households, water treatment purchasing water	Diarrhea (SR)	Stored water in household	organoleptic water quality,	No	Bivariate (Wilcoxon’s Signed Ranked test; Wilcoxon’s Ranked Sums test)	
13	Baker *et al.* (2013)	2007-2010	Case-control	children <5 years	4,096	Yes	Water-63	Time taken in fetching water, proportion with access to piped water	Purchasing water, Collecting water from alternative sources, water storage in households	Diarrhea (C)	Water points and stored water in household	faecal indicator organism test, free residual chlorine	No	Bivariate (Pearson chi-square tests, Fischer exact test, T-tests), Multivariate (logistic regression model)	Collecting water, continuous access to water, time taken to collect water, breastfeeding, both parents living at home, wealth quintile index, caretaker’s level of education
14	Kone- Coulibaly *et al.* (2010)	2008	Case- control	households	280	Yes	N/A	Unpredictable interruptions, proportion with access to piped water	Collecting water from alternative sources	Cholera (C), diarrhea (SR)	N/A	N/A	Yes	Multivariate (logistic regression models)	Contact with a diarrheal patient, experiencing unpredictable water interruptions, level of education, source of drinking water, attending a gathering, consuming hot food, consuming cold food, having received health education on cholera
15	Yilgwan *et al.* (2010)	2005	Cross- sectional individual	households	200	No	N/A	Proportion with access to piped water	Water treatment, collecting water from alternative sources	Diarrhea (SR)	N/A	N/A	No	Multivariate (logistic regression model)	Family size, Number of children in the household, educational status of household head, income level of household head, Domestic source of water, water treatment
16	Schaetti *et al.* (2013)	2008	Cross- sectional individual	respondents	356	No	N/A	NR	NR	Cholera (SR)	N/A	N/A	Yes	Bivariate (Wilcoxon test, Kruskal- Wallis test, Pearson chi-square test, Fischer exact test)	
17	Dunne *et al.* (2001)	1999	Case-control	Cases- households of women who attended HIV clinic	120	No	Water-120	Proportion with access to piped water, unpredictable interruptions	water storage in household, purchasing water, water treatment	Diarrhea (SR)	Stored water in household	faecal indicator organism test, free residual chlorine	No	NR	
18	Machdar *et al.* (2013)	2010	Cross- sectional individual	Households	110	No	Water-NR	Proportion with access to piped water, unpredictable interruptions	water storage in household, purchasing water, collecting water from alternative sources	rotavirus (SR), cryptosporidium (SR), diarrhea (SR)	Stored water in household	faecal indicator organism test, pathogen tests	No	Cost effective analysis	
19	Usman *et al.* (2005)	1995-2001	Cross- sectional ecological	Patients visiting health facilities	6,165	No	Water-NR	NR	Collecting water from alternative sources	Cholera (C)	Water points	faecal indicator organism test,	Yes		
20	Endris *et al.* (2019)	2016	Case- control	households	300	Yes	Stool-NR	NR	Collecting water from alternative sources, purchasing water	Cholera (CC)	N/A	N/A	Yes	Bivariate (Pearson chi-square test)	Source of water consumed, water treatment, level of hygiene/sanitation, type of food consumed, attended a gathering
21	Ubosi (2018)	2018	Cross- sectional individual	Mothers of infants <6 months	202	No	N/A	Proportion with access to piped water	Collecting water from alternative sources, purchasing water	Diarrhea (SR)	N/A	N/A	No	Bivariate (Pearson correlation coefficients, Chi-square test)	Exclusive breastfeeding, piped water supply
22	Blanton *et al.* (2015)	2010	Cross- sectional individual	households	39	No	Water-398	Proportion with access to piped water, scheduled water interruptions	Water storage in household, purchasing water, water treatment	Cholera (SR)	Water points and stored water in household	faecal indicator organism test, free chlorine residual	Yes	Bivariate (T-tests, Pearson chi-square tests, Wilcoxon rank-sum tests)	
23	Kuitcha *et al.* (2008)	2007	Cross- sectional individual	households	1,397	No	N/A	Proportion with access to piped water	Collecting water from alternative sources, water treatment, purchasing water	Dysentery (SR), diarrhea (SR), typhoid (SR)	N/A	N/A	No	Bivariate (Kruskal Wallis H-test)	
24	Yongsi (2010)	Survey: 2002; Microbiological & Medical Investigation: 2005 & 2008	Cross- sectional individual	children between 6-59 months	3,034	No	Water-508	Proportion with access to piped water	Collecting water from alternative sources	Diarrhea (SR)	Water points and Stored water in household	faecal indicator organism test, pathogen tests	No	Bivariate (Chi-square test,), spatial analysis	
25	Abaje *et al.* (2009)	2008	Cross- sectional individual	households	220	No	N/A	Proportion with access to piped water, scheduled interruptions, distance to the water source, L/P/D	Collecting water from alternative sources, purchasing water, water treatment	Cholera (SR), Typhoid (SR), Dysentery (SR)	N/A	N/A	N/A	Bivariate (Chi-square tests)	
26	Nkhuwa (2003)	Chemical and bacteriological analysis from water utility company: 1995-1997, 1999-2000; Health facility data: 1997 & 1999	Cross- sectional ecological	Patients visiting health facilities	1,864 in 1997, 6,219 in 1999	No	Water-14	Proportion with access to piped water	Collecting water from alternative sources, drilling boreholes	Cholera (C)	Water points	faecal indicator organism test, free chlorine residual, organoleptic water quality,	No	NR	
27	Sow *et al.* (1999)	1995-1996	Cross- sectional ecological	Cholera cases in health facilities	141 in 1995, 182 in 1996	No	N/A	Proportion with access to piped water	Purchasing water, collecting water from alternative sources	Cholera (C)	N/A	N/A	Yes	NR	
28	Julvez *et al.* (1998)	1995 (human samples), 1996 (water samples)	Cross- sectional individual	Households, children <10 years	322 people, 161 children	No	Water-15	L/P/D	Collecting water from alternative sources	Amoebiasis (SR)	Water points	faecal indicator organism test, pathogen test	No	NR	
29	Adane *et al.* (2017)	2014	Case- control	Children <5 years	760	Yes	Water- 192	scheduled interruptions, L/P/D,	water storage in households	Diarrhea (SR)	Stored water in household	faecal indicator organism test	No	Multivariate (Multiple logistic regression models)	
30	Akinbo *et al.* (2010)	2008-2009	Case- control	HIV-infected persons	500	No	Stool-500	Proportion with access to piped water	Collecting water from alternative sources	Cryptosporidium (CC), diarrhea (SR)	N/A	N/A	No	Bivariate (Pearson chi-square test)	
31	Nguyen *et al.* (2014)	2012	Case- control	Individuals >= 5 years	147	No	Water-80, Stool-30	Proportion with access to an improved source of water, scheduled interruptions	Water storage in households, purchasing water, illegal connections	Cholera (C)	Stored water in household	organoleptic water quality	Yes	Multivariate (Multiple logistic regression models), Bivariate (Wald Test)	Vended water, unsafe water, education level of household head, consuming hot food, consuming crab, consuming okra
32	Stoler *et al.* (2011)	2009-2010	Cross- sectional individual	Women, children	2093 women, 810 children	Yes	N/A	Scheduled water interruptions, proportion with access to piped water	Purchasing water, water storage in households	Diarrhea (SR)	N/A	N/A	No	Multivariate (multiple logistic regression models, random mixed effects models), Bivariate (ANOVA, chi-square test)	Mother’s self-reported overall health, purchased water as primary source of drinking water, daily bathroom expense, days of water rationing

^1^CC- Culture confirmed, C- Clinically confirmed, SR- self-reported NR-Not Reported; N/A- Not Available

### Connectedness of the study designs used

**Figure 3.**
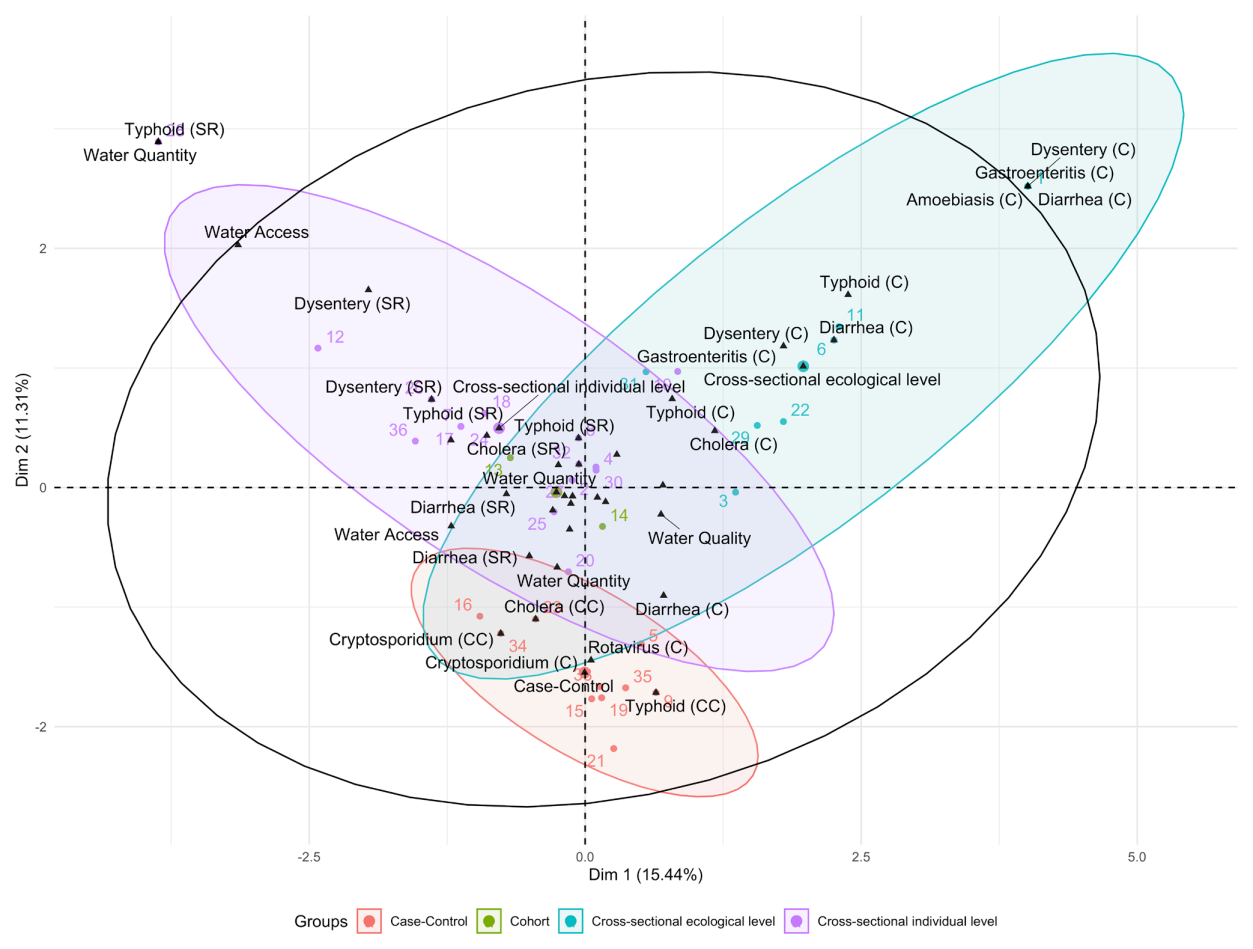
Included studies and study design types, plotted against the first two principal components derived from study design characteristics.

We assessed the connectedness in the study design methods used by the articles to understand the nexus between water sufficiency and health outcomes, as shown in Figure 3. The axes in the biplot represented the first two principal components of the input data which explained 27% of the total variability, showing weak correlation among the study designs.

The black triangle markers in Figure 3 represent the mean centres for the health outcomes and the characteristics of piped water supply that were studied by the articles. The correlation circle is portrayed by the uncolored hollow black circle. The colored confidence ellipses, which are plotted around the group mean points, represent the study design methods employed by the studies and the size of the ellipses are based on the variance of each group. The numbers represent each publication included in our study.

From this analysis, we observed that cross-sectional individual-level, cross-sectional ecological level and case control studies had a high variance and were the three commonly used study designs. Cross-sectional individual study designs were generally used in self-reported health outcomes while cross-sectional ecological and case control study designs were used in assessing clinically confirmed and culture confirmed health outcomes respectively. Water quantity and quality were mainly assessed using cross-sectional individual and ecological level study designs, whereas water access was mainly assessed using cross-sectional individual-level study designs. An unusual combination of self-reported typhoid and water quantity was observed as an outlier ( Figure 3). Use of cohort study designs in assessing the association between waterborne diseases and syndromes and water sufficiency was under-utilised.

## Discussion

Our study presents the results of a scoping review on associations between water supply and waterborne diseases and syndromes in large cities across Africa. We find that majority of the studies have been published since 2005. The relationship between piped water sufficiency and waterborne diseases/syndromes has mainly been studied using cross-sectional individual level study designs employing bivariate statistical methods. The main measures of water sufficiency used are access levels to piped water and water quality assessments while the health indicators mainly used are self-reported or clinically confirmed health outcomes. Cohort study design methods, measure of availability of piped water using quantifiable measures that include either per capita daily water consumption or water interruptions, cryptosporidium, cyclosporiasis, amoebiasis, rotavirus water borne diseases and culture confirmed assessment of health outcomes have been under-utilised. Similarly, multivariate methods which are important in assessing the confounders or alternative transmission pathways have been seldomly used.

Piped water has been listed as the primary source of improved water in this region ^66^, however results from this review contest to this with no evidence of sufficient piped water supply in the urban areas. Daily per capita water consumption and mode of access have been reported to be inversely proportional to the level of health concern, in outbreak and non-outbreak conditions ^21^. However, these two variables were under-studied and only assessed by two studies, neither of which investigated an outbreak ^51, 53^.

The use of alternative or secondary water sources, that are often unimproved (as classified by the Joint Monitoring Programme (JMP) of the World Health Organisation (WHO) and United Nation’s International Children’s Emergency Fund (UNICEF)), have been listed as one of the prevalent transmission pathways for water-related pathogens, due to high exposure to faecal contamination ^13, 67^. Adequate water treatment has the potential to reduce contamination of these water supplies by half ^43^. The studies included in this review reported use of alternative water sources as a key coping mechanism for poor or intermittent water supply while only a small proportion reported use of water treatment. Water contamination tests were a common assessment of water quality, contributing to the increased evidence of contamination in the predominant coping mechanisms employed by residents in urban areas.

Water storage, which was the second major coping mechanisms employed by the residents in urban areas, was observed as having the potential to increase the burden associated with waterborne diseases and syndromes. Low income earners, who account for 61% of the population in Africa, regularly practice poor water storage ^68, 69^. On the other hand, residents with a high income mainly invest in large storage tanks to ensure they enjoy safe storage and adequate water consumption even during periods of irregular water supply ^70^. The in-depth qualitative assessment of poor water storage practices and their association with waterborne diseases was under-studied. None of the studies focused on user reported organoleptic characteristics of stored water in their households.

Diarrhea and cholera were the majorly self-reported and clinically confirmed health outcomes respectively while cryptosporidium, cyclosporiasis, amoebiasis, rotavirus water borne diseases were under-studied. These four waterborne diseases are among the major etiological agents associated with moderate to severe diarrhea in children below five years ^9, 71^. Additionally, clinically and culture confirmed health outcomes are the two main approaches used in case definition of diseases of public health concern, with cases confirmed through objective assessment of samples at the laboratory ^72^. However, culturally confirmed health outcomes were seldomly employed in these studies, making it difficult to assess the public health burden associated with waterborne diseases.

Cross-sectional ecological and individual-level studies and case control studies were the main study designs used to understand the association between water sufficiency and health. Cohort study designs and multivariate statistical methods were under-utilised, limiting the detection of hotspots.

One of the limitations of our study was a lack of studies in Luanda, Kinshasa, Cairo, Johannesburg, Khartoum cities that had a population of more than 5 million people as at 2014 and are expected to be mega-cities by 2030 ^73^. Furthermore, there were no studies on cyclopsoriasis which was one of the waterborne diseases under our study criteria. Another limitation of our study was potential bias introduced through the choice of databases to conduct the search. Furthermore, we did not omit any studies based on the quality appraisal conducted on the included publications. These limitations have also been reported in other scoping reviews ^74^. The use of a non-conventional analysis method in our review may have also been a limitation assessing the connectedness of the study designs, health outcomes, water sufficiency and assessment of water quality. Similarly, our analysis methods deviated from the published protocol found here ^25^ where we had proposed to conduct cluster analysis to differentiate self-reported diarrheal diseases with etiological agents. This was not possible due to the diverging water sufficiency characteristics reported by the studies. We also did not present digital maps which overlayed the study locations and the water scarcity peer reviewed maps, as stated in the scoping review protocol. This is because the main outcome of our study was depicting under utilised study designs, health outcomes and water sufficiency metrics.

## Conclusion

Monitoring of health outcomes and the trends in availability and mode of access of piped water should be prioritised in urban areas in Africa in order to implement interventions towards reducing the burden associated with waterborne diseases and syndromes. This will contribute towards understanding the exposure pathways. Similarly, this is an area that can be used to assess the strategies of Africa being closer to achieving the United Nations SDGs regarding sustainable cities, adequate water, good health and wellbeing of its citizens and the Africa Union aspiration of having an African continent that is based on growth and sustainable development while coping with water insufficiency.

## Data availability

All data underlying the results are available as part of the article and no additional source data are required.

### Reporting guidelines

Open Science Framework. PRISMA-ScR reporting checklist for ‘The nexus between improved water supply and waterborne diseases in urban areas in Africa: a scoping review” DOI: https://doi.org/10.17605/OSF.IO/8TKSR ^75^

Data are available under the terms of the Creative Commons Attribution 4.0 International license (CC-BY 4.0).
