# Supplementary material for: The nexus between improved water supply and water-borne diseases in urban areas in Africa: a scoping review
Source: AAS Open Res. 2021 May 28;4:27. [Version 1] doi: 10.12688/aasopenres.13225.1 (PMC8311817; doi:10.12688/aasopenres.13225.1)
Supplement: Peer review history

# Peer review: Reviewer response for version 1

**DOI:** 10.21956/aasopenres.14345.r28652

**Published:** 2021-07-20

**Authors:** Prince Antwi-Agyei

**Affiliations:** 1 University of Energy and Natural Resources, Sunyani, Ghana

**Introduction:**

1. “The sub-Saharan Africa (SSA) has experienced the highest annual urban population growth rate (more than 3.5%) in the world”. *Which year or period is the growth rate being referenced to?*

**Methodology:**

1. Any justification why authors used 2014 as the baseline year for the literature search? Meanwhile, the results section presented studies which were conducted before 2014. Some clarity is needed on this.
2. Also, any reason why the authors selected cities with population more than 500,000 as of 2014 as part of the inclusion criteria? This has also been stated in the abstract.
3. Table 1: The study focuses on urban African cities and there are over 50 countries in Africa and so what informed the choice of the ‘search terms’ for the cities listed under population? More explanation is needed to clarify this.

**Results and discussion:**

1. “A total of 32 articles that assessed the association of water sufficiency in urban areas and waterborne diseases and syndromes in SSA were published between 1998 and 2019”. *Probably, the authors should provide some clarity because my understanding was that only studies from 2014 were included as part of the inclusion criteria in the methods section.*
2. Figure 1 shows that a total of 2619 articles were recorded from the search which is different from what was stated in the main text under study selection of the results section (3099). Try and reconcile the two.
3. It is unclear why authors did not consider the quality of the published studies as one of their inclusion criteria. This seems important and would have influenced the outcome of the scoping review and it is unclear how many of the final 32 studies were of poor quality. It would have been good for the authors to discuss the quality of the paper and link it to the strength of evidence these studies provided in terms of any associations between improved drinking water and water-borne diseases.

Are the rationale for, and objectives of, the Systematic Review clearly stated?

Yes

Is the statistical analysis and its interpretation appropriate?

Yes

Are sufficient details of the methods and analysis provided to allow replication by others?

Yes

Are the conclusions drawn adequately supported by the results presented in the review?

Partly

Reviewer Expertise:

Environmental Engineering, WASH, Solid waste management, faecal sludge management, Water quality, health risk assessment

I confirm that I have read this submission and believe that I have an appropriate level of expertise to confirm that it is of an acceptable scientific standard.

# Peer review: Reviewer response for version 1

**DOI:** 10.21956/aasopenres.14345.r28712

**Published:** 2021-07-12

**Authors:** Dickson W. Lwetoijera

**Affiliations:** 1 Environmental Health and Ecological Science Department, Ifakara Health Institute, Ifakara, Tanzania; 2 School of Life Sciences, Nelson Mandela African Institute of Science and Technology, Arusha, Tanzania

The reported scoping review is very detailed, and the provided methodology description is very detailed to allow replication. I do applaud the authors for jotting down a number of limitations that are important to consider, and they contextualized these finding across different cities in Africa. 

 In the review studies, the authors noted that only 8 out of 32 studies provided details on sample size calculation; owing to the importance of this variable, especially when studies focus on quantitative data collection; it is important for the authors to recognize this as one of the limitations. This is because the recorded findings/conclusion from included studies without sample size calculation details might have been either overstated or understated.

Are the rationale for, and objectives of, the Systematic Review clearly stated?

Yes

Is the statistical analysis and its interpretation appropriate?

Yes

Are sufficient details of the methods and analysis provided to allow replication by others?

Yes

Are the conclusions drawn adequately supported by the results presented in the review?

Partly

Reviewer Expertise:

Environmental Health Scientists, Medical entomologists,

I confirm that I have read this submission and believe that I have an appropriate level of expertise to confirm that it is of an acceptable scientific standard.
